# A comparative study of the self-assembly of achiral and chiral hairy nanoparticles with polystyrene cores and poly(2-hydroxyethylmethacrylate) hairs†

**DOI:** 10.1039/d0ra04951d

**Published:** 2020-10-08

**Authors:** Azza Habel, Ishrat M. Khan

**Affiliations:** Department of Chemistry, Clark Atlanta University Atlanta Georgia USA ikhan@cau.edu

## Abstract

Hairy nanoparticles with polystyrene cores (PS cores) and poly(2-hydroxyethylmethacrylate) (PHEMA) shells were synthesized by combining living anionic polymerization and atom transfer radical polymerization (ATRP). The structural characterization was carried out by FT-IR and NMR spectroscopy (^1^H NMR, ^13^C NMR, APT ^13^C NMR and ^1^H ^13^C HMQC). The thermal stability of the PS cores was not affected by grafting PHEMA on their surfaces. A differential scanning calorimetry (DSC) thermogram of the HNPs showed two distinct transition temperatures indicating microphase separation. Chiral HNPs were prepared by inducing chirality in the achiral HNPs by complexation with *R*- or *S*-mandelic acid. The circular dichroism (CD) spectroscopy of complexes of the HNPs/*R*- or *S*-mandelic acid indicated the formation of enantiomeric chiral structures. The self-assembled structures formed from the achiral HNPs show different surface morphologies, porous and zigzag, dependent on the solvents used. Blends of polystyrene functionalized with hydroxyl groups and PHEMA show different morphology and thermal properties compared with the core–shell HNP system. The chiral HNPs self-assembled into donut like structures or toroids with sizes in the range between 200 to 5000 nm. The study suggests that chirality can be utilized to develop interesting self-assembled structures.

## Abstract 2

Chiral hairy nanoparticles (HNP), prepared by complexation of achiral HNPs with *R*- or *S*-mandelic acid, self-assemble into toroidal or donut like structures.

## Graphical abstract

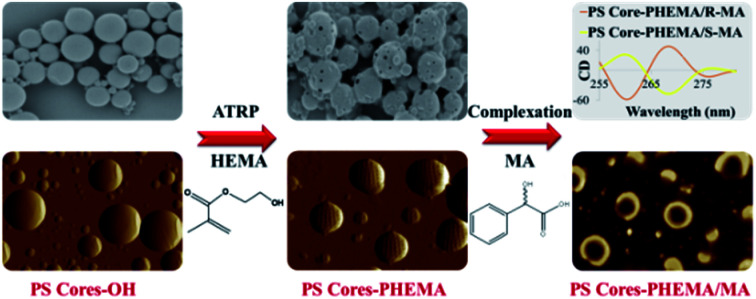

## 1 Introduction

More than a half-century after Feynman presented a talk entitled “There's Plenty of Room at the Bottom,” nanoscale research has been developing at an ever expanding rate.^1^ Nanoparticles with dimensions between 1 to 100 nm are among the most important focus within the area of nanotechnology. Nanoparticles have much better properties than the bulk materials and, in some cases, show novel properties. The synthesis, assembly, characterization, and applications of nanoparticles have been reported in detail.^2,3^ Hairy nanoparticles (HNPs) which fall under the category of nanoparticles can be broadly defined as comprising a core (inner material) and a shell (outer layer material). The synthesis of HNPs is of significant interest because of the possibility for constructing novel functional nanostructured materials. The inorganic core–shell nanoparticles have been of interest because they can exhibit superior properties arising from the combination of characteristics and properties of the core particles and polymer components.^4,5^ They possess unique chemical, physical, or biological properties due to their small size (10 nm to 100 nm), the increasing surface area to volume ratio and the presence of multiple functionalities on the shells, resulting in active materials for various areas of modern technology^6,7^ such as resistant coatings,^8,9^ pollution control, tire and rubber industry,^10^ catalysis,^11^ sensing, nanocarriers for drug and gene delivery.^12,13^ Nanoparticles with their unique multifunctional and self-assembling properties have been widely used as valuable building blocks with great potential. Molecular self-assembly can be across all length scales with different shapes.^14^ The assembly of building blocks which may comprise of atoms, molecules, nano- and microscale structures into macroscopic structures is an idea that runs through chemistry, biology and material science.^15^ Linking behavior of materials to the spatial arrangement of their fundamental building blocks are difficult to predict.^16^

At the nanoscale, the ability of polymeric nanoparticles to assemble to generate functional materials can lead to improved functional properties.^17^ The unique self-assembling property of block copolymers which can adopt a broad variety of morphologies, such as, lamellar, toroidal, cylindrical and spherical structures depending on the type and relative volume fractions of the blocks.^18–21^ Nanoparticles self-assembly process can be triggered by environmental factors including temperature, pH or solvent polarity in a broad range of applications.^22^ Structure–property relationships of copolymers continue to be an important topic for polymer chemists and physicists. Due to the development of polymerization techniques and combination of polymerization methods, copolymers which show interesting shapes have been reported.^23^ The progress in ionic polymerization techniques, in particular anionic polymerization of vinyl monomers, has resulted in well-defined polymers with controlled chain end functionalities, thereby modifying the surface properties of the particles.^24,25^ The development of ATRP as the most often used controlled/living radical polymerization has allowed the synthesis of well-defined block and graft copolymers which can be utilized as building blocks for new assemblies.^26,27^

The synthesis, design and characterization of nanoporous materials have been of interest due to their wide range of applications in biosensing,^28,29^ catalysis,^30,31^ drug delivery,^32^ and optics.^33^ Most of these applications require control in the pore size, functional membrane surface, chemical and thermal stability.^34,35^ It has been demonstrated that an amphiphilic phase separated copolymer of PS-*b*-PHEMA synthesized by ATRP has the ability to self-assemble into honeycomb patterned porous membranes.^36^ From the perspective of functional bionanomaterial engineering, the PS-*b*-PHEMA copolymers are of significant interest. Okano and co-worker reported that the copolymer showed excellent antithrombogenic properties *in vitro* examination.^37^ Hairy nanoparticles with polystyrene cores have shown interesting self-assembled structures. Zhou *et al.* synthesized HNPs, polystyrene cores with polydimethylsiloxane hairs. The obtained HNPs self-assembled into hierarchical suprastructures.^38^ If the production of a material results in the improvement of performance with a cheaper delivery of service, that material becomes more attractive and profitable. According to numerous studies, the complexation of polymers with chiral materials to get chiral polymeric structures has been achieved.^39–41^ These compounds with interesting assemblies and chiral properties may be used in numerous potential applications, such as, recognition, chiral sensor and chiral separation, as chiral stationary phases for liquid chromatography.^42,43^ Chiral separation is a great need for the production of pharmaceuticals and biomolecules because enantiopure molecules may have preferred biological activity compared to racemic mixtures of pharmaceutical molecules.^44,45^ Again, if polymer materials interacts with non-covalent interactions, *e.g.* blending polymer, there can be a significant improvement in the properties as well as creating special self-assemblies. For instance, a new self-assembly structure of a polymer blend which possesses potential applications both in drug delivery and water treatment have been reported. The self-assembly was studied by transmission electron microscopy.^46^ Lastly, the research area of core/shell nanoparticles have continued to produce breakthrough discoveries because of the synthesis, properties, modification, and applications of this new class of nanomaterials.

In this study, we have successfully synthesized the PS core/PHEMA shell nanoparticles by coupling polymerization methods. Living anionic polymerization was used to synthesize cross-linked polystyrene cores functionalized with hydroxyl groups and atom transfer radical polymerization (ATRP) was then carried out to prepare PHEMA hairs following the grafting form technique. The obtained structures were investigated by Fourier transform infrared spectroscopy (FT-IR), nuclear magnetic resonance (NMR), thermal gravimetric analysis (TGA), differential scanning calorimetry (DSC), dynamic light scattering (DLS), scanning electron microscopy (SEM) and atomic force microscopy (AFM). Complexes of the synthesized HNPs and PS core-OH, with *S*- and *R*-mandelic acids were prepared and the resulting chiral structures were studied by using circular dichroism (CD) spectroscopy. The morphology of the chiral and achiral HNPs was studied by AFM. Also, the preparation of a polymer blend of PS core-OH and PHEMA was carried out to compare its morphology and its thermal properties with the synthesized HNPs. The innovation of this study is that the novel chiral HNPs have advantage as the stationary phase in chiral chromatography because of its significantly higher surface to volume ratio compared to linear polymeric packing materials.

## 2 Experimental

### 2.1. Materials

*sec*-Butyllithium (*sec*-BuLi, 1.4 M in cyclohexane), 2-bromopropionyl bromide, calcium hydride (CaH_2_), copper(i) bromide (CuBr), benzophenone, sodium metal (Na), *N*,*N*,*N*′,*N*′′,*N*′′-pentamethyldiethylenetriamine (PMDETA), *S*-mandelic acid (*S*-MA), *R*-mandelic acid (*R*-MA), were purchased from Sigma-Aldrich Company and used as received. The solvents *N*,*N*-dimethylformamide (DMF), methanol (MeOH), chloroform (CHCl_3_), and diethyl ether were of analytical grade and purchased from Fisher Chemical Co. and were used as received. A mixture of styrene (S, Aldrich) and divinylbenzene (DVB, Aldrich) (90 S/10 DVB mol%) was stirred over calcium hydride for 24 h. The mixture was deoxygenated by three freeze–pump–thaw cycles, on the vacuum line, and then distilled into ampoules equipped with break-seals. The same procedures were applied to purifying propylene oxide (PO, Aldrich). Tetrahydrofuran (THF, Aldrich) was purified by refluxing over Na with benzophenone as indicator and distilled on the vacuum line into ampoules with break-seals. All ampoules were flame-sealed, and then stored in the freezer until needed. Monomer 2-hydroxyethyl methacrylate (HEMA, 99%, Aldrich) was purified twice by passing it over a short column filled with basic alumina to remove the inhibitor. Triethylamine (TEA, Aldrich) was stirred over calcium hydride for about 24 h and then distillation under reflux was done prior to use in reaction.

### 2.2. Methods

FT-IR spectra were recorded on KBr plates in transmission mode on a PerkinElmer Spectrum 65 FT-IR spectrometer. NMR spectra were recorded at room temperature on Bruker AVANCE III 500 MHz and Bruker AVANCE 400 MHz spectrometers. Tetramethylsilane (TMS, *δ* = 0) was used as an internal chemical shift standard. ^1^H NMR splitting abbreviations: s (singlet), m (multiplet) and br (broad). The hydrodynamic diameter was determined using DLS technique (DynaPro Nanostar Wyatt Technology Instrument). TGA was done with a TA-Q50 instrument from 25 °C to 600 °C. Samples were placed in platinum pans and heated in a nitrogen atmosphere at a heating rate of 10 °C min^−1^. DSC measurements were performed using a TA Instruments Q2000 differential scanning calorimeter under N_2_ at a heating rate of 10 °C min^−1^. CD measurements were performed using a JASCO-710 spectropolarimeter and quartz cuvette at room temperature. SEM images were taken on an Agilent Technologies 8500FE-SEM instrument with a 1.0 kV accelerating voltage and an upper secondary electron detector. The samples were prepared by spin-coating on a silicon wafer followed by air drying and coated with a layer of gold under vacuum *via* direct sputtering using a Technics Hummer Sputter Coater. AFM images were obtained using a Bruker Dimension FastScan Atomic Force Microscope. The AFM samples were prepared either by drop-casting or spin-coating in different solvents on a silicon wafer followed by air-drying.

### 2.3. Synthesis of poly(2-hydroxyethyl methacrylate) (PHEMA) by ATRP

2-Hydroxyethyl methacrylate (5 mL, 41 mmol) was placed in a 25 mL flask, followed by adding DMF (8 mL), CuBr (34 mg, 0.24 mmol), and PMDETA (0.05 mL, 0.24 mmol). The flask was then sealed and degassed with bubbling nitrogen for 50 min. 2-Bromopropionyl bromide (0.05 mL, 0.48 mmol), which was degassed with bubbling nitrogen for at least 40 min, was added to the reaction flask *via* a nitrogen purged hypodermic syringe. The mixture was stirred for 10 min and the flask was transferred to an oil bath maintained at 70 °C and stirred for 22 hours. The reaction mixture was then quenched *via* exposure to air and dilution with methanol. The resultant polymer solution was filtered through a column filled with silica to remove the copper complex. The polymer was obtained by precipitation of the reaction mixture into diethyl ether, and then drying the product in a vacuum at room temperature. The yield obtained was 1.0 g. PHEMA: ^1^H NMR (DMSO-d_6_, 500 MHz): *δ* [ppm] 0.68–1.17 (tacticity splitting, 3H), 1.47–2.00 (tacticity splitting, 2H), 3.57 (br s, 2H), 3.88 (br s, 2H), 4.82 (br s, 1H). ^13^C NMR (DMSO-d_6_, 125 MHz): *δ* 16.6–18.9, 44.5–45.6, 50.8–54.2, 59.0, 66.7, 176.8–177.9. *T*_g_ = 50 °C (Fig. S1 and S2†).

### 2.4. Synthesis of polystyrene core functionalized with hydroxyl groups (PS core-OH)

The reaction was performed in a glass reactor equipped with a magnetic stirrer connected to the vacuum line. The break-sealed ampoules containing the mixture of monomers (S and DVB), PO, and THF were attached to the reactor. The break seal ampoule with the THF (20 mL) was crushed to add the solvent to the reactor that already reached high vacuum, and then the break seal ampoule with the mixture of styrene (1.50 g, 14.40 mmol) and divinylbenzene (0.22 g, 1.68 mmol) was broken to add the monomers to THF in the reactor. The mixture was cooled down with an isopropyl alcohol/liquid nitrogen bath to −78 °C. Under vigorous stirring, *sec*-BuLi (1.4 M) in cyclohexane (1 mL) was rapidly injected into the reactor using a glass syringe fitted with a stainless-steel needle at −78 °C. By addition of the initiator, the color of the reaction mixture turned to reddish orange. The polymerization reaction was conducted at −78 °C for 1.5 h with continuous stirring. In the second step of the reaction, the propylene oxide (0.40 mL, 5.71 mmol) ampoule was smashed open by breaking the break-seal and the reaction mixture was gradually warmed to room temperature. The colour of the solution quickly changed to yellow and became lighter with time. The reaction was then carried out at room temperature for 15 h with continuous stirring. Termination of the reaction was achieved by injecting 0.60 mL of a mixture of methanol/hydrochloric acid, 37% (5 : 1 v/v%). The core particles were obtained as a white powder by precipitation into methanol, and further dried in a vacuum oven at room temperature to give 1.25 g of core particles. ^1^H NMR (CDCl_3_, 400 MHz): *δ* [ppm] 0.49–0.90 (6H), 0.91–1.62 (5H, 2HPS core), 1.63–2.91 (3H, H_PS core_), 3.67 (br s, 1H), 6.29–7.40 (H_aromatic PS core_).^13^C NMR (CDCl_3_, 125 MHz): *δ* [ppm] 10.6–11.6, 14.1–16.0, 22.1–24.7, 28.4–30.2, 31.6–31.9, 39.3–41.1, 41.6–46.6, 64.9–66.0, 125.6–127.9, 145.3–146.1. Decomposition TGA (N_2_) = ∼434 °C. *T*_g_ (DSC) = 116 °C.

### 2.5. Synthesis of bromoester end-functionalized polystyrene core (PS core-Br)

In a 100 mL round flask connected to vacuum line, PS core-OH (0.1 g) was placed along with a magnetic stirrer bar, and then the flask was evacuated and refilled three times with nitrogen. Distilled THF (20 mL) was injected through the rubber septum, followed by addition of TEA (0.20 mL, 1.43 mmol) and then 2-bromopropionyl bromide (0.15 mL, 1.43 mmol) was added dropwise to the reaction mixture. The reaction was left to stir for 23 h at room temperature. The resulting polymer was precipitated into methanol, and then dried in a vacuum for 24 h at room temperature. The yield obtained was 85 mg. ^1^H NMR (CDCl_3_, 500 MHz): *δ* [ppm] 0.49–0.90 (6H), 0.91–1.62 (5H, 2H_PS core_), 1.63–2.91 (6H, H_PS core_), 4.14 (br s, 1H), 4.38 (br s, 1H), 6.29–7.40 (H_aromatic PS core_).

### 2.6. Synthesis of HNP with polystyrene core and PHEMA hairs (PS core–PHEMA)

PS core-Br (25 mg) was placed into a 100 mL flask, CuBr (0.19 mg, 0.0014 mmol), PMDETA (0.24 mg, 0.0014 mmol), HEMA (31 mg, 0.24 mmol) and DMF (7 mL) were added. The flask was closed and subjected to three cycles of freeze–pump–thaw to remove oxygen. The room temperature mixture was stirred and placed in a 70 °C oil bath for 24 h. The solvent was removed by air flow and the product was obtained by precipitation into methanol and dried in a vacuum oven overnight at room temperature to give 37 mg of the target hairy nanoparticles. ^1^H NMR (CDCl_3_, 500 MHz): *δ* [ppm] 0.49–1.18 (12H), 1.21–1.62 (5H, 2H_PS core_), 1.63–2.91 (3H, 2H_PHEMA_, H_PS core_), 3.78 (2H_PHEMA_), 4.14 (br s, 1H, 2H_PHEMA_), 6.29–7.40 (H_aromatic PS core_). ^13^C NMR (CDCl_3_, 125 MHz): *δ* [ppm] 10.9–11.4, 14.1–16.0, 16.6–18.7,19.9–20.9, 21.6, 28.4–30.2, 31.6–31.9, 33.2, 37.1, 39.3–41.1, 41.6–47.6, 59.5, 65.7, 69.2, 125.6–127.9, 145.3–146.1.

### 2.7. Preparation PS core-OH/PHEMA blend

The PS core-OH/PHEMA blend was prepared by dissolving the polymers (1 : 1 w/w) in DMF and stirring for 24 h at room temperature. The solvent was allowed to evaporate slowly by air flow and the solid mixture was dried in a vacuum oven for 24 h.

## 3 Results and discussion

**Scheme 1:**
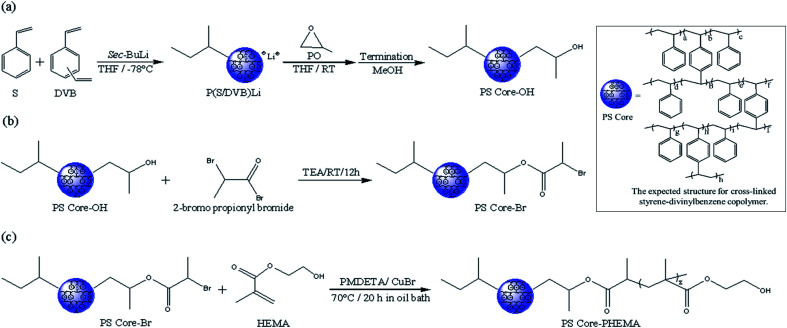
Synthesis of HNPs (PS core–PHEMA).

Scheme 1 outlines the synthetic procedures for the target HNPs. First, living anionic polymerization was used to synthesize cross-linked polystyrene cores functionalized with hydroxyl groups. Next, the hydroxyl groups of PS core-OH were modified into the bromoester groups. Finally, the PHEMA polymer chains were grafted from PS core-Br nanoparticles *via* ATRP. This procedure yielded core–shell nanoparticles with PS cores and outer layer of covalently attached PHEMA hairs.

### 3.1. Spectroscopic characterization of PS core-OH, PS core-Br, and PS core-PHEMA

**Fig. 1:**
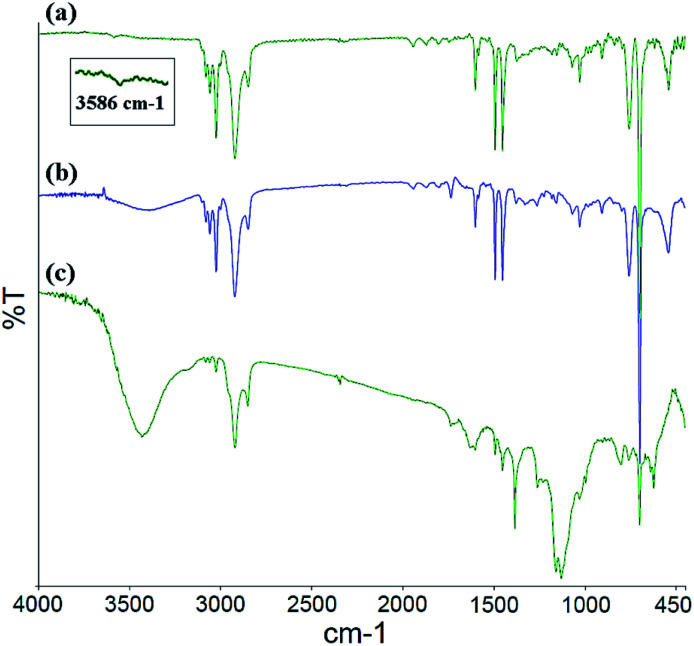
FT-IR spectra of (a) PS core-OH, (b) PS core-Br, and (c) PS core–PHEMA.

The FT-IR spectra for the PS core-OH, PS core-Br, and PS core–PHEMA are shown in Fig. 1. In all the spectra, a multipeak at 1452, 1493, and 1601 cm^−1^ (the aromatic C=C stretching modes) is observed, as well as peaks in the ranges of 2900–3000 cm^−1^ and of 3000–3090 cm^−1^ attributed respectively to the C–H aliphatic and C–H aromatic vibrations. The FT-IR spectrum of PS core-OH (Fig. 1a) shows a characteristic absorption band at 3586 cm^−1^ corresponding to –OH stretching. Fig. 1b indicates the existence of characteristic adsorption bands of PS core-Br. In comparison with the FT-IR spectrum of PS core-OH (Fig. 1a), new absorption bands are observed at 1735 cm^−1^ and at 1261 cm^−1^ corresponding to C=O and C–O–C in the ester bond, respectively. After ATRP with HEMA, the new stretching bands appeared in ranges of 3250–3600 cm^−1^and of 1020–1300 cm^−1^, assigned to –OH and C–O of PHEMA hair, respectively (Fig. 1c). A range of 1727–1735 cm^−1^ assigned to all ester carbonyl stretching (C=O) bands is observed in the FT-IR spectrum of PS core–PHEMA.

**Fig. 2:**
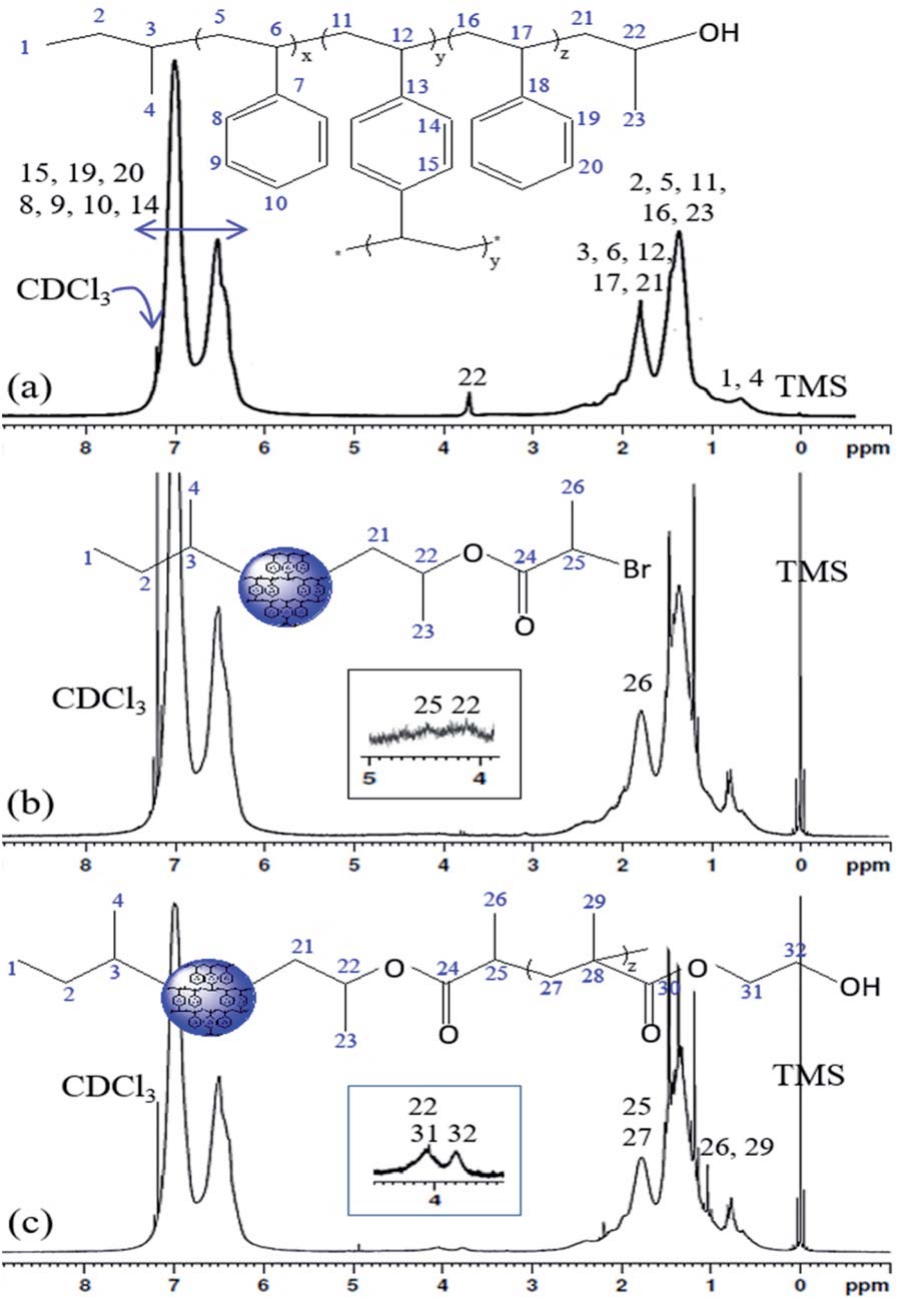
^1^H NMR spectra of (a) PS core-OH, (b) PS core-Br, and (c) PS core–PHEMA in CDCl_3_.

The ^1^H NMR spectra of the PS core-OH, PS core-Br, and PS core–PHEMA are shown in Fig. 2. The PS core-OH spectrum (Fig. 2a) shows a characteristic peak centered at 3.67 ppm corresponding to methine proton bonded to carbon atom next to the terminal hydroxyl group. Protons of the polystyrene backbone are observed in the range from 0.91 to 2.94 ppm, and the aromatic protons are observed in the range from 6.29 to 7.40 ppm. The peak at 0.74 ppm is assigned to the methyl protons of the *sec*-butyl group at the chain ends. It can be clearly seen that there are no signals at 5.15 to 5.65 ppm assigned to methylene protons (CH_2_=) of unreacted vinyl groups for the cross-linker DVB. This indicated that all vinyl groups of the cross-linkers were copolymerized and resulted in the formation of cross-linked polystyrene core particles. The ^1^H NMR spectrum for PS core-Br particles (Fig. 2b) shows a peak at 4.38 ppm which can be assigned to the methine proton of bromoester group. Compared with the ^1^H NMR spectrum of PS core-OH, the signal for methine proton at 3.67 ppm shifted to 4.14 ppm for PS core-Br, indicating that the esterification reaction has been performed completely. The presence of PHEMA brushes in the particle can be confirmed by the appearance of the characteristic signals in the ^1^H NMR spectrum of PS core–PHEMA (Fig. 2c) at 3.78 and 4.14 ppm attributed to –CH_2_–OH and –CH_2_–OC=O– groups of the PHEMA block, respectively. The signal at 4.14 ppm (attributable to –CH_2_–OC=O– groups of the PHEMA block) is assigned to methine group of propylene segment adjacent to the carboxylate groups. The missing signal at 4.38 ppm in ^1^H NMR spectrum of PS core–PHEMA, assigned to the methine proton of bromoester segment, shifted upfield and overlapped with protons of the backbone chain of copolymer as a result of disappearance of electron withdrawing bromine. ^13^C NMR spectroscopy was used to analyze the regiochemistry of the nucleophilic ring-opening of propylene oxide and for the possibility of oligomerization of the propylene oxide chain end.

**Fig. 3:**
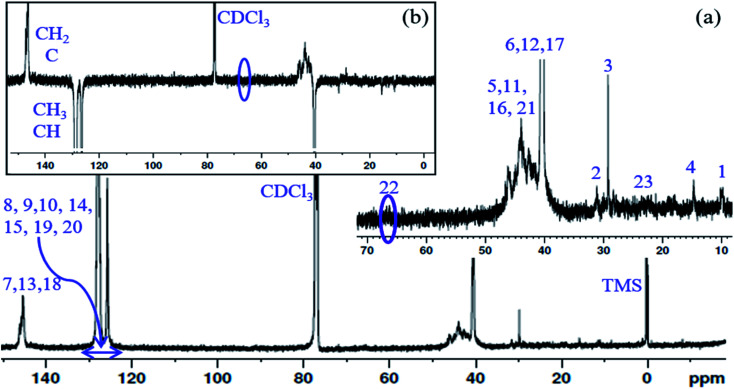
(a) ^13^C NMR spectrum of PS core-OH in CDCl_3_, and (b) APT ^13^C NMR spectrum of PS core-OH in CDCl_3_.

^13^C NMR spectrum of the PS core-OH and APT ^13^C NMR spectrum of PS core-OH are shown in Fig. 3. The ^13^C NMR spectrum of PS core-OH (Fig. 3a) shows one weak peak in the region of 65–71 ppm (64.9–66.0 ppm) that is assigned to the carbons bonded to oxygen and this indicates the absence of oligomerization of propylene oxide. The peaks in the 22–25 ppm range correspond to the methyl carbon resulting from attack of the polystyrene organolithium core (P(S/DVB)Li) at the least hindered carbon to form a secondary alcohol chain end functional group. For further evidence of this conclusion, APT ^13^C NMR experiment was carried out on the PS core-OH sample. It can be observed from Fig. 3b that the spectral region that contains the signal from the functional chain-end carbons shows only inverted phase peak, indicating absence of any methylene-type carbons within this spectral region. Fig. S3† shows the ^13^C NMR spectrum of PS core–PHEMA, and the peaks can be assigned as represented in Table 1.

**Table 1:** Observed ^13^C NMR chemical shift for PS core–PHEMA

Carbon #	*δ* [ppm]	Carbon #	*δ* [ppm]
1	10.9–11.4	22	69.2
2	28.4–30.2	23	19.9–20.9
3	31.6–31.9	24, 30	Not observed
4	14.1–16.0	25	33.2
5, 11, 16	41.6–47.6	26	21.6
6, 12, 17	39.3–41.1	27	41.6–47.6
7, 13, 18	145.3–146.1	28	37.1
8, 9, 10,19, 20	125.6–127.9	29	16.6–18.7
14, 15	125.6–127.9	31	65.7
21	45.3	32	59.5

**Fig. 4:**
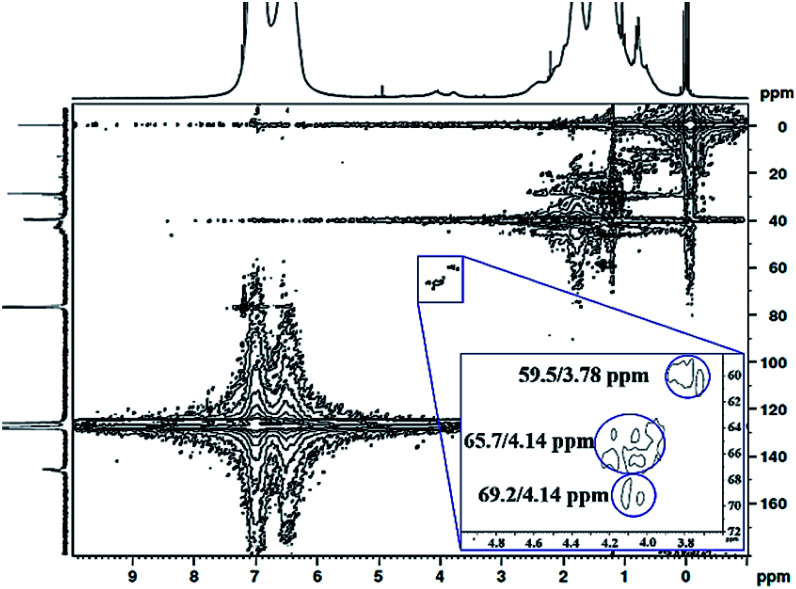
^1^H ^13^C HMQC NMR spectrum of PS core–PHEMA in CDCl_3_.

The ^1^H ^13^C heteronuclear multiple quantum coherence (^1^H ^13^C HMQC) correlation spectrum for PS core–PHEMA is shown in Fig. 4. The spectrum shows one-bond correlation peaks assigned to the three weak carbon signals at 59.5, 65.7, and 69.2 ppm, attributed to methylene carbons in PHEMA (–CH_2_–OH and –CH_2_–OC=O–) and methine carbon of propylene segment adjacent to the carboxylate groups, respectively, and their protons centered at 59.5/3.78, 69.2/4.14, and 65.7/4.14 ppm. The result is that the methine proton of propylene segment is overlapped with the methylene protons (–CH_2_–OC=O–) of PHEMA block at 4.14 ppm.

### 3.2. Thermal behaviour

**Fig. 5:**
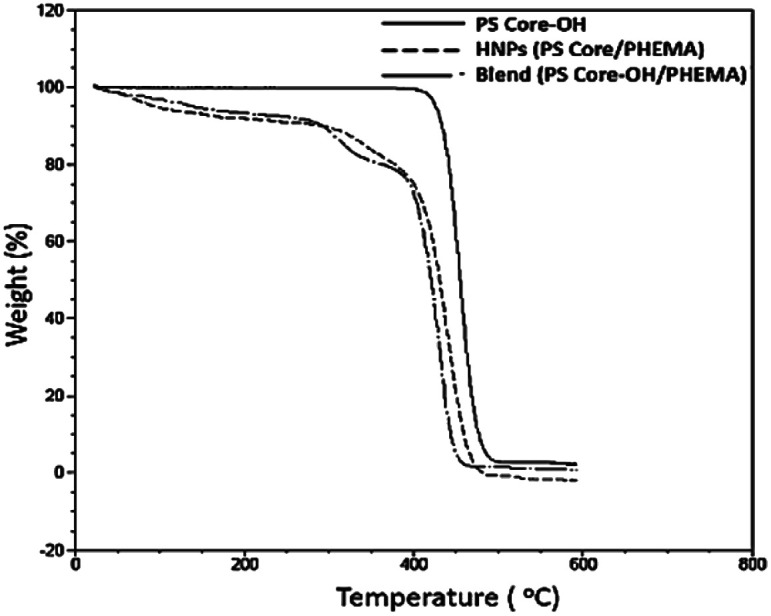
TGA thermograms of PS core, PS core–PHEMA, and blend.

TGA was employed to study the thermal stability of all synthesized nanoparticles (Fig. 5). The thermal decomposition behaviour of the synthesized HNPs between 20 °C and 600 °C shows a two-step mass loss process corresponding to a two-phase morphology. The onset of the first weight loss at about 308 °C corresponds to the decomposition of the PHEMA shell component and the second weight loss commences at about 416 °C corresponding to the decomposition of the PS core particles. The PS nanoparticles with PHEMA hairs displayed a slightly lower thermal stability than that of PS core-OH particles which began to degrade at ∼434 °C. The DSC results for the HNP (PS core–PHEMA), PS core-OH and PHEMA are shown in Fig. S4.† The HNPs showed two clearly separated transitions corresponding to a *T*_g_ for the PS phase at 107 °C and a *T*_g_ for the PHEMA phase at 63 °C, indicating phase separation. As compared with *T*_g_s for PHEMA and PS core-OH, the lowering of the *T*_g_ of PS phase and higher *T*_g_ of PHEMA in the HNPs are attributed to the interplay between the PS and PHEMA segments.

### 3.3. Morphology of the achiral HNPs

**Fig. 6:**
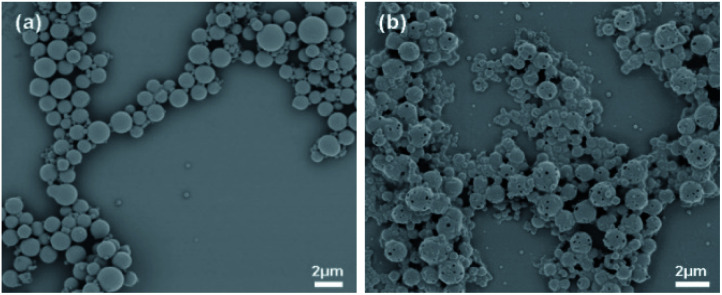
SEM images of samples prepared by spin-coating dispersions of 1 mg mL^−1^ in THF/MeOH (5 : 1 v/v%) mixture and coated with gold: (a) PS core-OH, and (b) PS core–PHEMA.

**Fig. 7:**
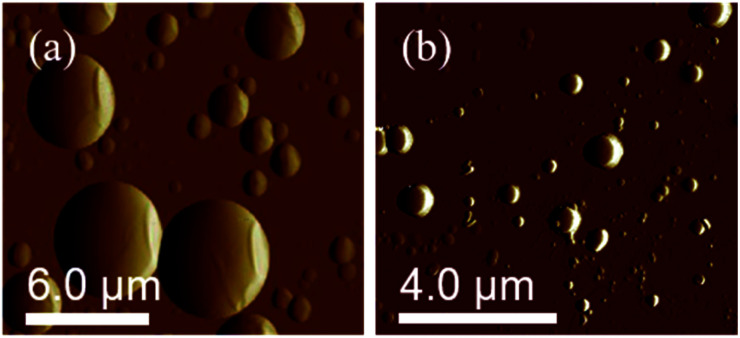
AFM images of PS core-OH prepared by drop-casting dispersions of 1 mg mL^−1^ (a) in THF, and (b) in DMF.

The SEM images show the surface morphology of the PS core-OH and the synthesized HNPs (Fig. 6). The PS core-OH nanoparticles (Fig. 6a) showed spherical morphologies with smooth surfaces compared with surfaces of unfunctionalized polystyrene cores particles that are porous.^41^ After grafting of PHEMA on the surfaces of PS core-OH, the HNPs (Fig. 7b) showed multiple-sized spherical morphology with porous surfaces.

**Fig. 8:**
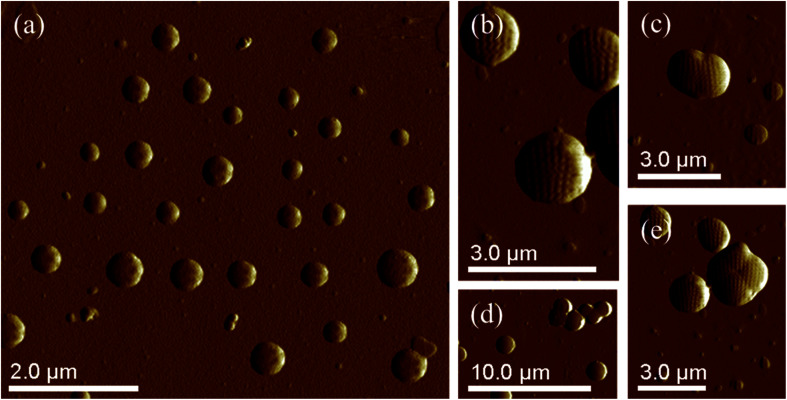
AFM images of self-assembly of PS core–PHEMA prepared by drop-coating dispersion of 1 mg in 1 mL DMF (a) and (b). AFM images of supra-assembly of HNPs: group of particles with 1.15 μm in diameter (c), groups of particles with 1.2 μm in diameter (d), and group of particles with 1.3 μm in diameter (e).

**Fig. 9:**
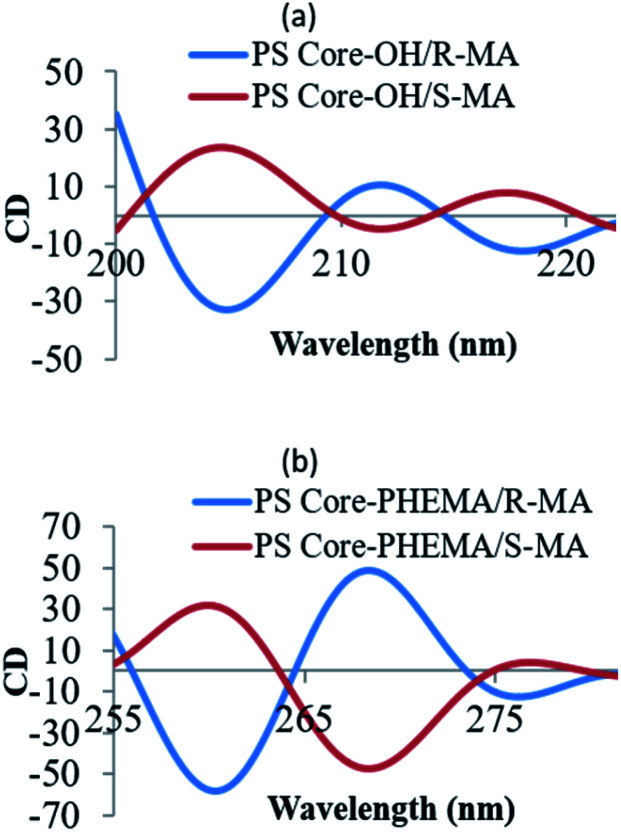
CD spectra of complexes of (a) PS core-OH/*R*- and *S*-MA, and (b) PS core–PHEMA/*R*- and *S*-MA.

The morphology of PS core–PHEMA was also investigated by AFM. The AFM images of the PS-core-OH in different solvents (Fig. 7a and b) clearly show spherical particles with smooth surfaces and this result is in good agreement with the SEM image as shown in Fig. 6a. The size of the particles was also measured by AFM, and the diameter of the particles was found to be in the range between 73 nm and 5480 nm. The smaller particles plausibly combine or fuse together to form the larger particles. DMF was used as solvent to prepare the HNP samples in order to study the surface. Spherical structures with zigzag surfaces of PS core–PHEMA are recognized as shown in Fig. 9a and b. The PHEMA hairs on the core surfaces perhaps self-assembled to form zigzag shells. The pure PS core-OH do not show the formation of the zigzag structures on the surfaces. The sizes of obtained HNPs were found to be in the range between 63 nm and 1.39 μm. The PS core–PHEMA particles were able to connect and fuse together to form supra assembly spherical particles with zigzag surfaces. The fusing of two particles, three particles, and four particles to form larger particles are observed in the AFM images (Fig. 8c–e).

### 3.4. Particle size distribution

The size distribution profiles of PS core and PS core–PHEMA particles are shown in Fig. S5a.† Compared to that of PS core-OH (Fig. S5b†), the average hydrodynamic radius, *R*_h_, for HNPs in CHCl_3_ increased from 50 to 66 nm, which should be attributed to the grafted PHEMA.

### 3.5. Morphology of the chiral HNPs

The CD spectral studies were used to investigate the induced chirality to PS core-OH and PS core–PHEMA prepared by complexation with chiral molecules, *R*- and *S*-MA. Induction of chirality into poly(3-methyl-4-vinylpyridine) by *R*- and *S*-MA has been reported.^40^Fig. 9a shows the CD spectra of PS core-OH/*R*-MA and *S*-MA complexes (4 mg PS core-OH: 7 mg MA: 4 mL DMF at room temperature). The complexes showed mirror image Cotton effect signals at 204.6, 211.8 and 218 nm, suggesting formation of enantiomeric structures. Further, the CD spectra of the complexes of obtained HNPs with the optically active mandelic acids (4 mg PS core–PHEMA: 7 mg MA: 4 mL DMF at room temperature) show three couples of positive and negative Cotton effect signals around 260, 268, and 277 nm (Fig. 9b) and this result indicated that the induction of chirality into obtained HNPs through noncovalent interaction with chiral compounds (*R*- and *S*-MA) is possible in DMF.

**Fig. 10:**
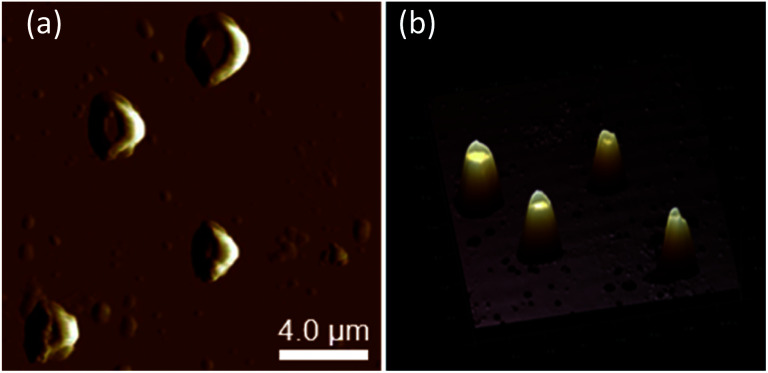
AFM images of PS core-OH/*S*-MA complex: (a) 2D, and (b) 3D.

**Fig. 11:**
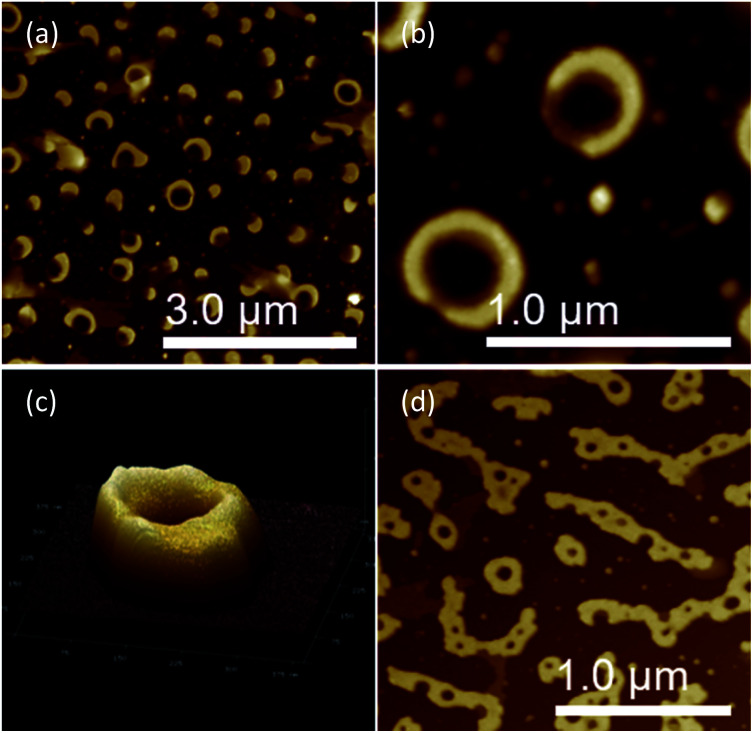
AFM images of PS core–PHEMA/*S*-MA complex prepared by drop-casting dispersion in CHCl_3_ (1 mg PS core–PHEMA: 1 mg *S*-MA: 1 mL CHCl_3_): (a and b) 2D, (c) 3D, and (d) PS core–PHEMA/*S*-MA complex prepared by a second drop of CHCl_3_ sample solution drop-casted.

**Fig. 12:**
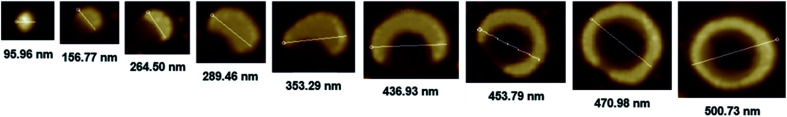
AFM images showing development of toroidal self-assembled structure of PS core–PHEMA/*S*-MA complex prepared by drop-casting dispersion in CHCl_3_ (1 mg PS core–PHEMA: 1 mg *S*-MA: 1 mL CHCl_3_).

The morphology of chiral complexes was studied by AFM. The two-dimensional (2D) AFM image of PS core-OH/*S*-mandelic acid complex (Fig. 10a) shows cone-like morphology, *i.e.* the structure is a valley surrounded by hills. The cones are at a height of ∼864.7 nm as shown in the three-dimensional (3D) AFM image (Fig. 10b). The complex of PS core–PHEMA and *S*-MA was self-assembled into donut like structures with size in the range between 200 nm to 5000 nm as shown in Fig. 11a and b. Such self-assembled structures are called toroids. From the 3D AFM images of HNPs/*S*-MA complex, the heights of toroid-like structures were measured to be in the range between 46 nm to 58 nm. As example, Fig. 11c shows the 3D AFM image of a toroidal structure with height 54 nm, inner diameter 75 nm, and outer diameter 224 nm. Interestingly, when the local particle concentration is very low (*i.e.* 1 drop of drop casted CHCl_3_ sample solution on the silicon wafer), the toroidal particles are not connected or are separated (Fig. 11a and b). When the local particle concentration was increased (*i.e.* a second drop of CHCl_3_ sample solution drop-casted on the first drop), the structures show supra assembly (Fig. 11d), groups of toroidal morphologies, which are not continuous but broken up into long and short islands. By checking the AFM image in Fig. 11a and b, the development of the toroidal structures is observed. Fig. 12 summarizes the development of 492 nm toroidal self-assembled structure.

The observed morphology of the chiral HNPs compared to the achiral HNPs clearly suggest that chirality can be used as an element of control to obtain well defined self-assembled structures. In the chiral HNP one has the shape control of the HNPs plus the element of chirality which most likely impart an additional control during the self-assembly process.

### 3.6. PS core-OH/PHEMA blend

To understand the role of the well-defined HNP structure on the physical properties and in the formation of the self-assembled structures, the PS core-OH/PHEMA blend was also studied. The thermal stability of the PS core-OH/PHEMA blend (15% wt PHEMA) was investigated by means of TGA from 20 to 600 °C in nitrogen atmosphere at a heating rate of 10 °C min^−1^ and the result compared with TGA for HNPs (PS core–PHEMA) as shown in Fig. 5. A distinct two step degradation process is observed for the blend sample. The onset of the first weight loss corresponds to the decomposition of the PHEMA shell components and the second weight loss is attributed to the decomposition of the PS cores. The PHEMA segments in the blend began to degrade at ∼288 °C and the PS cores segments at 408 °C. As compared with TGA result of the obtained HNPs, the components of HNPs displayed higher thermal stability than in the blend. This higher in thermal stability is most likely because of the covalent bond connecting the two components in the HNPs whereas in the blend the two components or phases are held together by weaker hydrogen bonds. Furthermore, the core–shell system is highly organized system compared to the blend system. In the HNPs, the PHEMA hairs extend orderly away from the cores surfaces and that can result both in chain entanglement and formation of inter-chain hydrogen bonding.

**Fig. 13:**
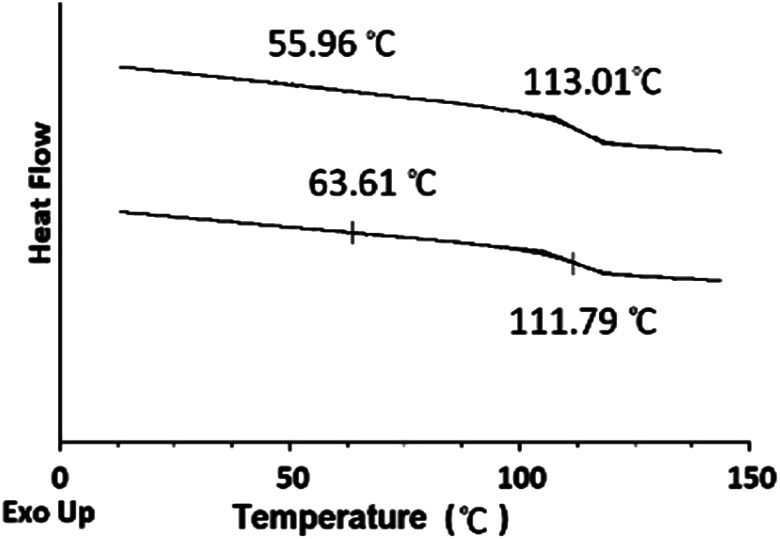
DSC thermograms of PS core-OH/PHEMA blends.

The DSC thermograms for the blends of different compositions are summarized in Fig. 13. The blends show two glass-transitions, one for the PHEMA and the other for the PS core-OH, which suggests the formation of a phase separated system. Both pure PHEMA and pure PS core-OH exhibit only one *T*_g_ at 50 °C and 116 °C, respectively (Fig. S4†). The PHEMA phase *T*_g_ shows a slight increase due to the mixing of the two blend components at the interphase. The increase in *T*_g_ for PHEMA can be attributed to polystyrene particles impeding the segmental mobility of the PHEMA chains by interacting with the chains. Similarly, the decrease of the higher *T*_g_ phase may also be attributed to mixing of the two components with the lower *T*_g_ PHEMA induces a quicker onset of the segmental motion.

**Fig. 14:**
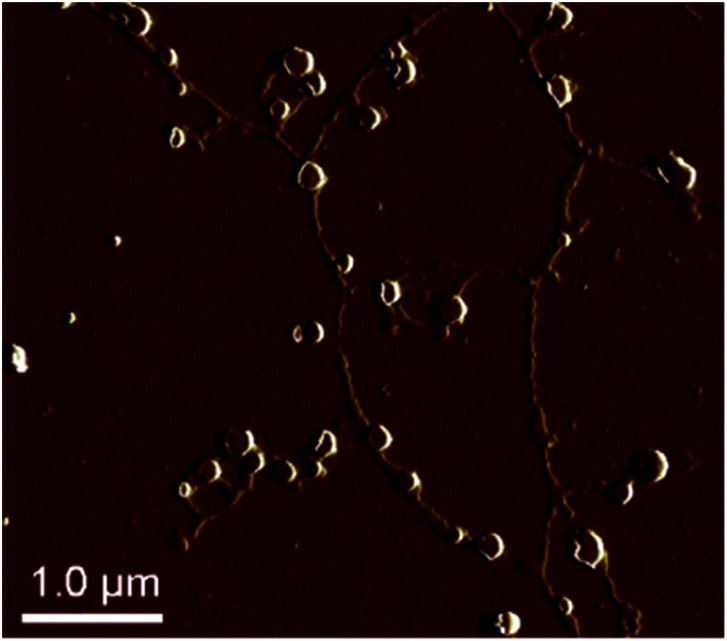
AFM image of PS core-OH/PHEMA blend (1 : 1 w/w%).

The morphology of a PS core-OH/PHEMA blend (1 : 1 w/w%) was studied by AFM. Fig. 14 shows the AFM images of the blend sample prepared in THF/DMF (1 : 1 v/v%) mixture and spin-coated on a silicon wafer. The AFM image of the blend shows network structures, *i.e.* the structure is spherical particles and connected string like structures. It is clear that the HNPs show quite different self-assembled structures with the shape and the composition playing a vital role in formation of the structures.

## 4 Conclusions

In summary, we have successfully synthesized phase separated polymeric HNPs consisting of PS cores and PHEMA hairs by combining living anionic polymerization and ATRP techniques. PS cores were *in situ* formed by living anionic copolymerization of styrene and divinylbenzene, and then propylene was reacted with living cores to functionalize their surfaces by hydroxyl groups. Next, PHEMA hairs were grafted on the polystyrene core nanoparticles surfaces. FT-IR, 1D NMR and 2D NMR were used to confirm the structure of the obtained HNPs. TGA curves show that the grafting of PHEMA did not thermally affect the PS cores stability because of the ease of breakdown the ester bonds. The two glass transitions were clearly seen in the DSC thermograms of the obtained HNPs and this indicates the presence of a hydrophobic PS core phase and a hydrophilic PHEMA shell phase in the HNPs. As observed by AFM and SEM images, solvents have significant effect on the surface morphologies of hairy nanoparticles. Porous surfaces, which were observed using mixture of methanol and THF. The achiral HNPs showed zigzag surfaces when DMF was used as dispersing solvent and this suggests that the hair chains perhaps were assembled to form zigzag structures on the surfaces. The AFM image of PS core-OH and PHEMA blend shows spherical structures connected by string like structures. The chiral HNPs were prepared by complexation of the achiral HNPs with *R*- or *S*-mandelic acid. The chiral HNPs self-assembled into donut like structures or toroids with sizes in the range between 200 to 5000 nm. The study suggest that chirality can be utilized to develop interesting self-assembled structures.

## Conflicts of interest

There are no conflicts to declare.

## Supplementary Material

RA-010-D0RA04951D-s001

## References

[cit1] Feynman R. (1960). There's Plenty of Room at the Bottom. Eng. Sci..

[cit2] Chaudhuri R. G., Paria S. (2012). Core/Shell Nanoparticles: Classes, Properties, Synthesis Mechanisms, Characterization, and Applications. Chem. Rev..

[cit3] Burda C., Chen X., Narayanan R., El-sayed M. A. (2005). Chemistry and Properties of Nanocrystals of Different Shapes. Chem. Rev..

[cit4] Mohan S. J., Mohan E. C., Yamsani M. R. (2009). One-Pot Synthesis of Hairy Nanoparticles by Emulsion ATRP. Macromolecules.

[cit5] Chen C., Zhang T., Zhu L., Zhao B., Tang P., Qiu F. (2016). Hierarchical Superstructures Assembled by Binary Hairy Nanoparticles. ACS Macro Lett..

[cit6] Fernandes N. J., Company D. C., Koerner H., Base W. A. F., Giannelis E. (2013). Hairy Nanoparticle Assemblies as One-Component Functional Polymer Nanocomposites: Opportunities and Challenges. MRS Commun..

[cit7] Wang X., Hall J. E., Warren S., Krom J., Magistrelli J. M., Rackaitis M., Bohm G. G. A. (2007). Synthesis, Characterization, and Application of Novel Polymeric Nanoparticles. Macromolecules.

[cit8] Yezek L., Scha W., Chen Y., Gohr K., Schmidt M. (2003). Influence of Hair Density and Hair Length on Interparticle Interactions of Spherical Polymer Brushes in a Homopolymer Matrix. Macromolecules.

[cit9] Klos J., Pakula T. (2003). Interaction of a Spherical Particle with Linear Chains. II. Chains End-Grafted at the Particle Surface. J. Chem. Phys..

[cit10] Wang X., Foltz V. J., Rackaitis M., Bohm G. G. A. (2008). Dispersing Hairy Nanoparticles in Polymer Melts. Polymer.

[cit11] Helms B., Guillaudeu S. J., Xie Y., Mcmurdo M., Hawker C. J., Frøchet J. M. J. (2005). One-Pot Reaction Cascades Using Star Polymers with Core-Confined Catalysts. Angew. Chem., Int. Ed..

[cit12] Siegwart D. J., Whitehead K. A., Nuhn L., Sahay G., Cheng H., Jiang S., Ma M., Lytton-Jean A., Vegas A., Fenton P., Levins C. G., Love K. T., Lee H., Cortez C., Collins S. P., Li Y. F., Jang J., Querbes W., Zurenko C., Novobrantseva T., Langer R., Anderson D. G. (2011). Combinatorial Synthesis of Chemically Diverse Core-Shell Nanoparticles for Intracellular Delivery. Proc. Natl. Acad. Sci. U. S. A..

[cit13] Von-Werne T., Patten T. E. (1999). Preparation of Structurally Well-Defined Polymer−Nanoparticle Hybrids with Controlled/Living Radical Polymerizations. J. Am. Chem. Soc..

[cit14] McManus J. J., Charbonneau P., Zaccarelli E., Asherie N. (2016). The Physics of Protein Self-Assembly. Curr. Opin. Colloid Interface Sci..

[cit15] Radjabian M., Abetz C., Fischer B., Meyer A., Abetz V. (2017). Influence of Solvent on the Structure of an Amphiphilic Block Copolymer in Solution and in Formation of an Integral Asymmetric Membrane. ACS Appl. Mater. Interfaces.

[cit16] Glotzer S. C., Solomon M. J., Kotov N. A. (2004). Self-Assembly: From Nanoscale to Microscale Colloids. AIChE J..

[cit17] Al-Rehili S., Fhayli K., Hammami M. A., Moosa B., Patil S., Zhang D., Alharbi O., Hedhili M. N., Möhwald H., Khashab N. M. (2017). Anisotropic Self-Assembly of Organic-Inorganic Hybrid Microtoroids. J. Am. Chem. Soc..

[cit18] Onses M. S., Ramırez-Hernandez A., Hur S., Sutanto E., Williamson L., Alleyne A. G., Nealey P. F., Pablo J. J., Rogers J. A. (2014). Block Copolymer Assembly on Nanoscale Patterns of Polymer Brushes Formed by Electrohydrodynamic Jet Printing. ACS Nano.

[cit19] Hamley I. W. (2003). Nanotechnology with Soft Materials. Angew. Chem., Int. Ed..

[cit20] Chiu J. J., Kim B. J., Yi G., Bang J., Kramer E. J., Pine D. J. (2007). Distribution of Nanoparticles in Lamellar Domains of Block Copolymers. Macromolecules.

[cit21] Subramani S., Khraisat A., George A. (2008). Self-Assembly of Proteins and Peptides and their Applications in Bionanotechnology. Curr. Nanosci..

[cit22] Grzelczak M., Vermant J., Furst E. M., Liz-marza L. M. (2010). Directed Self-Assembly of Nanoparticles. ACS Nano.

[cit23] Wang G., Hu B., Fan X., Zhang Y., Huang J. (2012). Synthesis of Amphiphilic Tadpole-Shaped Copolymers by Combination of Glaser Coupling with Living Anionic Polymerization and Ring-Opening Polymerization. J. Polym. Sci., Part A: Polym. Chem..

[cit24] Ueda K., Hirao A., Nakahama S. (1990). Synthesis of Polymers with Amino End Groups. 3. Reactions of Anionic Living Polymers with α-Halo-ω-aminoalkanes with a Protected Amino Functionality. Macromolecules.

[cit25] Takenaka K., Hirao A., Nakahama S. (1995). Synthesis of End-Functionalized Polymers by means of Living Anionic Polymerization, 3. Synthesis of Polystyrene and Polyisoprene with 1,3-Butadienyl Termini by Reaction of their Anionic Living Polymers with 6-Bromo-3-Methylene-1-Hexene. Macromol. Chem. Phys..

[cit26] Carlmark A., Malmstro E. E. (2003). ATRP Grafting from Cellulose Fibers to Create Block-Copolymer Grafts. Biomacromolecules.

[cit27] V Tsarevsky N., Matyjaszewski K. (2007). “Green” Atom Transfer Radical Polymerization: From Process Design to Preparation of Well-Defined Environmentally Friendly Polymeric Materials. Chem. Rev..

[cit28] Hotta K., Yamaguchi A., Teramae N. (2012). Deposition of Polyelectrolyte Multilayer Film on a Nanoporous Alumina Membrane for Stable Label-Free Optical Biosensing. J. Phys. Chem. C.

[cit29] Deng J., Toh C.-S. (2013). Impedimetric DNA Biosensor Based on a Nanoporous Alumina Membrane for the Detection of the Specific Oligonucleotide Sequence of Dengue Virus. Sensors.

[cit30] Li Q., Cui S., Yan X. (2012). Electrocatalytic Oxidation of Glucose on Nanoporous Gold Membranes. J. Solid State Electrochem..

[cit31] Wei L., Kawamoto K. (2013). Upgrading of Simulated Syngas by Using a Nanoporous Silica Membrane Reactor. Chem. Eng. Technol..

[cit32] Jeon G., Yang S. Y., Kim J. K. (2012). Functional Nanoporous Membranes for Drug Delivery. J. Mater. Chem..

[cit33] Zhao Q., Yin M., Zhang A., Prescher S., Antonietti M., Yuan J. (2013). Hierarchically Structured Nanoporous Poly(Ionic Liquid) Membranes: Facile Preparation and Application in Fiber-Optic pH Sensing. J. Am. Chem. Soc..

[cit34] Alothman Z. A. (2012). A Review: Fundamental Aspects of Silicate Mesoporous Materials. Materials.

[cit35] Khabibullin A., Fullwood E., Kolbay P., Zharov I. (2014). Reversible Assembly of Tunable Nanoporous Materials from “Hairy” Silica Nanoparticles. ACS Appl. Mater. Interfaces.

[cit36] Wu B., Zhu L., Ou Y., Tang W., Wan L., Xu Z. (2015). Systematic Investigation on the Formation of Honeycomb-Patterned Porous Films from Amphiphilic Block Copolymers. J. Phys. Chem. C.

[cit37] Okano T., Aoyagi T., Kataoka K., Abe K., Sakurai Y., Shimada M., Shinohara I. (1986). Hydrophilic-Hydrophobic Microdomain Surfaces Having an Ability to Suppress Platelet Aggregation and their in Vitro Antithrombogenicity. J. Biomed. Mater. Res..

[cit38] Zhou G., Person V., Khan I. M. (2016). Hairy Nanoparticles with Hard Polystyrene Cores and Soft Polydimethylsiloxane Shells: One-Pot Synthesis by Living Anionic Polymerization and Characterization. Macromol. Chem. Phys..

[cit39] Sannigrahi B., McGeady P., Khan I. M. (2004). Helical Poly(3-methyl-4-vinylpyridine)/Amino Acid Complexes: Preparation, Characterization, and Biocompatibility. Macromol. Biosci..

[cit40] Ortiz L. J., Pratt L., Smitherman K., Sannigrahi B., Khan I. M. (2002). Helical and Higher Structural Ordering in Poly(3-methyl-4-vinylpyridine). ACS Symp. Ser..

[cit41] Yashima E., Matsushima T., Okamoto Y. (1997). Chirality Assignment of Amines and Amino Alcohols Based on Circular Dichroism Induced by Helix Formation of a Stereoregular Poly((4-carboxyphenyl) acetylene) through Acid-Base Complexation. J. Am. Chem. Soc..

[cit42] Maeda K., Maruta M., Shimomura K., Ikai T., Kanoh S. (2016). Chiral Recognition Ability of an Optically Active Poly(diphenylacetylene) as a Chiral Stationary Phase for HPLC. Chem. Lett..

[cit43] Blaschke G. (1971). Chromatographic Resolution of Racemates. Angew. Chem., Int. Ed. Engl..

[cit44] Mohan S. J., Mohan E. C., Yamsani M. R. (2009). Chirality and its Importance in Pharmaceutical Field - An Overview. Int. J. Pharm. Sci. Nanotechnol..

[cit45] Sekhon B. S. (2010). Enantioseparation of Chiral Drugs - An Overview. Int. J. ChemTech Res..

[cit46] Hanying Z., Jie G., Ming J., Yingli A. A. (1999). A New Approach to Self-Assembly of Polymer Blends in Solution. Polymer.

