# Supplementary material for: A comparative study of the self-assembly of achiral and chiral hairy nanoparticles with polystyrene cores and poly(2-hydroxyethylmethacrylate) hairs
Source: RSC Adv. 2020 Oct 8;10(61):37358–68. doi: 10.1039/d0ra04951d (PMC9057163; doi:10.1039/d0ra04951d)
Supplement: RA-010-D0RA04951D-s001 [file RA-010-D0RA04951D-s001.pdf]

Electronic Supplementary Material (ESI) for RSC Advances.
This journal is © The Royal Society of Chemistry 2020

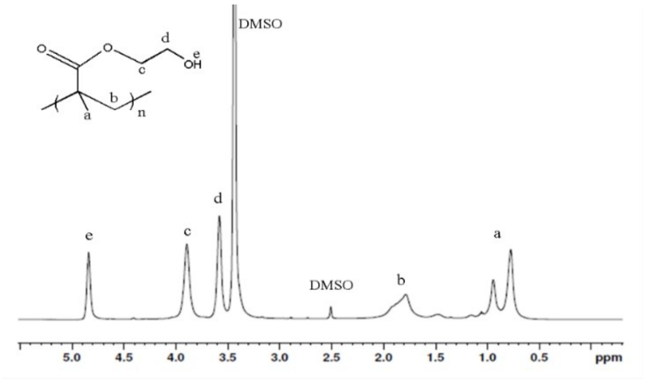

Fig. S1. ^{1}H NMR spectrum of PHEMA in DMSO-d_6.

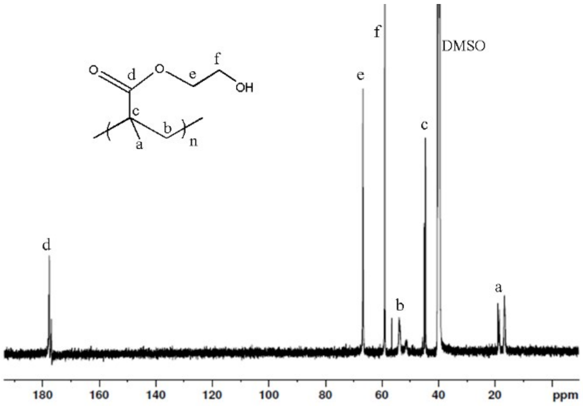

Fig. S2. ^{13}C NMR spectrum of PHEMA in DMSO-d_6.

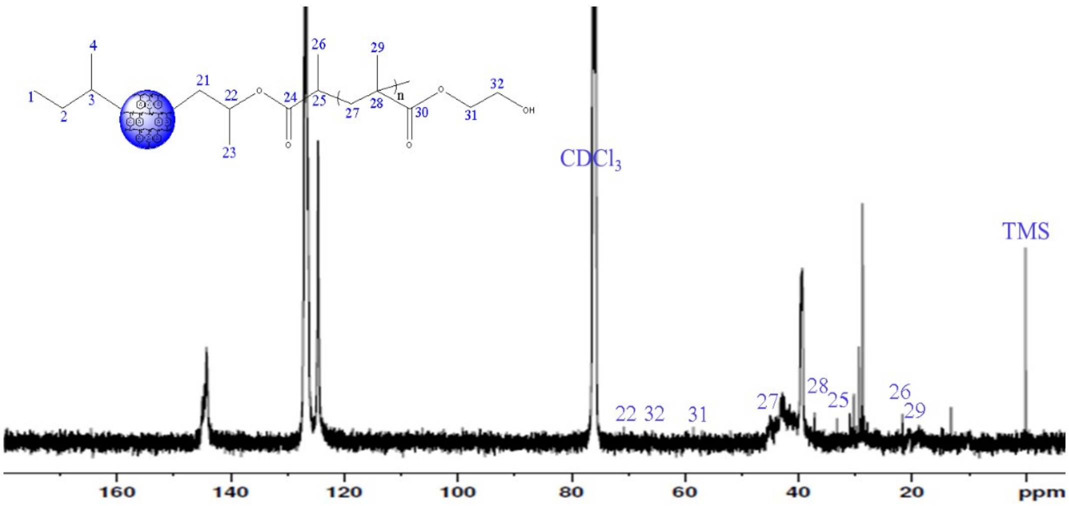

Fig. S3 ^{13}C NMR spectrum of PS Core-PHEMA in $CDCl_3$.

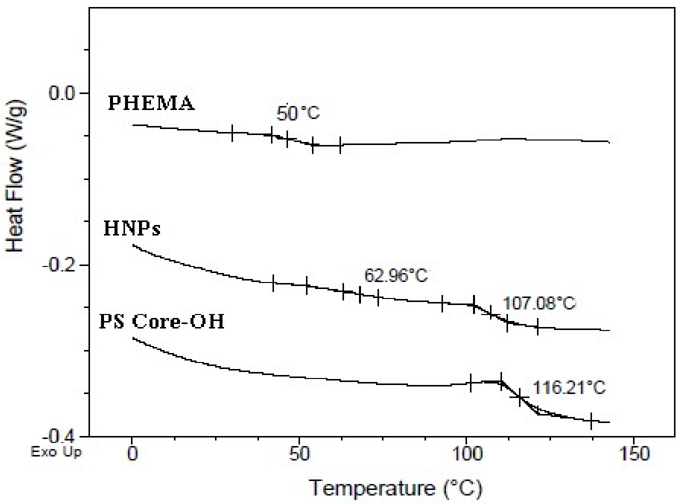

Fig. S4. DSC thermograms of HNPs (PS Core-PHEMA), PS Core-OH, and PHEMA.

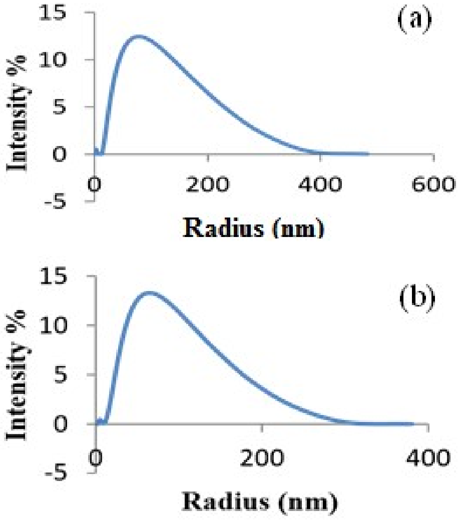

Fig. S5. The particles size distribution profiles in $CHCl_3$ of (a) PS Core-PHEMA, and (b) PS Core-OH.
